# Human BBB-on-a-chip reveals barrier disruption, endothelial inflammation, and T cell migration under neuroinflammatory conditions

**DOI:** 10.3389/fnmol.2023.1250123

**Published:** 2023-09-25

**Authors:** Arya Lekshmi Nair, Linda Groenendijk, Roos Overdevest, Tania M. Fowke, Rumaisha Annida, Orsola Mocellin, Helga E. de Vries, Nienke R. Wevers

**Affiliations:** ^1^MIMETAS BV, Oegstgeest, Netherlands; ^2^Amsterdam UMC, Vrije Universiteit Amsterdam, Amsterdam Neuroscience – Neuroinfection and Neuroinflammation, Amsterdam, Netherlands

**Keywords:** blood–brain barrier, neuroinflammation, organ-on-a-chip, BBB-on-a-chip, transendothelial migration

## Abstract

The blood-brain barrier (BBB) is a highly selective barrier that ensures a homeostatic environment for the central nervous system (CNS). BBB dysfunction, inflammation, and immune cell infiltration are hallmarks of many CNS disorders, including multiple sclerosis and stroke. Physiologically relevant human *in vitro* models of the BBB are essential to improve our understanding of its function in health and disease, identify novel drug targets, and assess potential new therapies. We present a BBB-on-a-chip model comprising human brain microvascular endothelial cells (HBMECs) cultured in a microfluidic platform that allows parallel culture of 40 chips. In each chip, a perfused HBMEC vessel was grown against an extracellular matrix gel in a membrane-free manner. BBBs-on-chips were exposed to varying concentrations of pro-inflammatory cytokines tumor necrosis factor alpha (TNFα) and interleukin-1 beta (IL-1β) to mimic inflammation. The effect of the inflammatory conditions was studied by assessing the BBBs-on-chips’ barrier function, cell morphology, and expression of cell adhesion molecules. Primary human T cells were perfused through the lumen of the BBBs-on-chips to study T cell adhesion, extravasation, and migration. Under inflammatory conditions, the BBBs-on-chips showed decreased trans-endothelial electrical resistance (TEER), increased permeability to sodium fluorescein, and aberrant cell morphology in a concentration-dependent manner. Moreover, we observed increased expression of cell adhesion molecules and concomitant monocyte adhesion. T cells extravasated from the inflamed blood vessels and migrated towards a C-X-C Motif Chemokine Ligand 12 (CXCL12) gradient. T cell adhesion was significantly reduced and a trend towards decreased migration was observed in presence of Natalizumab, an antibody drug that blocks very late antigen-4 (VLA-4) and is used in the treatment of multiple sclerosis. In conclusion, we demonstrate a high-throughput microfluidic model of the human BBB that can be used to model neuroinflammation and assess anti-inflammatory and barrier-restoring interventions to fight neurological disorders.

## Introduction

The blood–brain barrier (BBB) is a protective physiological and metabolic barrier that ensures a homeostatic environment for healthy CNS functioning by strictly regulating movement of ions, molecules, and cells from the circulation to the brain (Abbott et al., 2010; Obermeier et al., 2013; Keaney and Campbell, 2015; Terstappen et al., 2021; Andjelkovic et al., 2023). Specialized brain endothelial cells form a tight barrier and express inter-endothelial adherens junction (AJ) and tight junction (TJ) proteins. The barrier function of the cerebral blood vessels is further supported by perivascular cells, such as pericytes and astrocytes, which contribute to low paracellular permeability of the BBB (Stamatovic et al., 2016; Jiang et al., 2018). Specific transporter systems at the BBB endothelial cells selectively allow entry of essential nutrients, such as glucose, to the brain while restricting harmful circulating pathogens and other neurotoxic molecules from accessing the brain (Shen and Zhang, 2010).

The protective nature of the BBB is altered in neurological diseases, for example in stroke and multiple sclerosis (MS), leading to neuroinflammation and contributing to disease progression (Zlokovic, 2008; Daneman, 2012; Daneman and Prat, 2015; Brambilla, 2019). Under neuroinflammatory conditions, several changes occur at the level of the BBB microvasculature allowing recruitment of leukocytes from the circulation. These include enhanced expression of cell adhesion molecules (CAMs), release of pro-inflammatory cytokines and chemokines, and reduced expression of BBB junctional proteins (Engelhardt and Ransohoff, 2012; Marchetti and Engelhardt, 2020; Profaci et al., 2020; Wang et al., 2023). In neuroinflammation, peripheral immune cells cross an inflamed BBB through a multi-step cascade of interconnected events leading to transendothelial migration (TEM). TEM is a dynamic and sequential process that involves an initial tether of leukocytes onto the BBB endothelial cell surface. Following the initial contact, the leukocytes roll and establish a firm adhesion before crawling and transmigrating into the brain parenchyma (Drevets and Leenen, 2000; Engelhardt and Ransohoff, 2012; Romo-González et al., 2012; Liebner et al., 2018).

The firm attachment of leukocytes to BBB endothelium is a critical step in the migratory process and is orchestrated by integrins on leukocytes, such as very late antigen-4 (VLA-4) and leukocyte function associated antigen-1 (LFA-1), and their respective endothelial ligands, vascular cell adhesion molecule 1 (VCAM-1) and intercellular adhesion molecule 1 (ICAM-1) (Engelhardt and Ransohoff, 2012). This is highlighted by the beneficial effects of Natalizumab, an anti-VLA4 humanized monoclonal antibody that is clinically approved for treating relapsing forms of MS (Coisne et al., 2009; Marchetti and Engelhardt, 2020). Natalizumab acts by binding to the α4-integrin subunit of VLA-4 on leukocytes, thereby blocking their interaction with VCAM-1 on BBB endothelial cells. Studies using intravital microscopy have shown that Natalizumab inhibits the firm attachment and subsequent transmigration of human T cells across the inflamed BBB in an experimental autoimmune encephalomyelitis (EAE) model, which is the most commonly used animal model of MS (Coisne et al., 2009).

Animal models are frequently used to study neuroinflammatory disorders. Although animal models have proven valuable, these models do not fully reflect the complex pathogenesis, clinical course, disease heterogeneity, and the altered immune homeostasis as seen in patients, impairing clinical development of novel therapeutic compounds (Mestas and Hughes, 2004; Gold et al., 2006; Simmons et al., 2013; Bjelobaba et al., 2018; Lassmann, 2020). Additionally, extrapolation of findings from experimental animal studies to humans has proven difficult. For example, the EAE model does not mirror the complex neuropathology and altered immune status as seen in MS patients and lacks cortical damage. Moreover, the impairment of the BBB during EAE differs from that seen in patients in which lesions come and go and during which inflammation may become compartmentalized behind a closed BBB (Lassmann and Bradl, 2017; Lassmann, 2019). Inter-species variation and differences in immune system responses between animals and humans thus lead to limited translation of results from pre-clinical animal studies to human clinical trials. Furthermore, animal models are relatively costly and time-consuming, hold ethical concerns, and allow only limited control over experimental conditions (Mestas and Hughes, 2004; O'Brown et al., 2018). To bridge this gap, *in vitro* models were developed, and have undergone significant advancement over the last decades. An example of these advancements is the use of induced pluripotent stem cell (iPSC) derived *in vitro* models, which allow disease modeling on a patient-specific level.

Transwell-based BBB models have been widely used to study TEM, often employing transfected or immortalized cell lines (Callahan et al., 2004; Mahad et al., 2006; Ubogu et al., 2006; Man et al., 2012; Daniels et al., 2013; De Laere et al., 2017; Sonar et al., 2017; Labus et al., 2018; Hawke et al., 2020; Meena et al., 2021; Ford et al., 2022). Others have used primary brain endothelial cells or stem cell-derived cells to study TEM (Bramley et al., 2017; Wimmer et al., 2019; Clé et al., 2020) in the Transwell system. In this platform, brain endothelial cells are often cultured on top of a porous, semi-permeable membrane that separates the top and bottom compartments of the Transwell. Immune cells are added to the top compartment and their migration across the endothelial cell monolayer towards a chemokine trigger in the bottom compartment is studied (Callahan et al., 2004; Mahad et al., 2006; Ubogu et al., 2006; Man et al., 2008). However, to closely mimic the *in vivo* situation and allow real-time imaging of TEM, *in vitro* models employing a membrane-free setup are more beneficial (Takeshita and Ransohoff, 2012; De Haan et al., 2021). Additionally, incorporation of fluid flow is crucial in *in vitro* modeling as shear forces promote leukocyte-endothelial interactions and transmigration (Cinamon et al., 2001a,b; Schreiber et al., 2007).

Dynamic *in vitro* BBB models such as microfluidic models, are better alternatives to study TEM. Microfluidic platforms comprise of small chips with micro-channels that enable (co-)culture of cells and miniaturized tissue constructs in a three-dimensional (3D) fashion under fluid flow. These models allow precise control over medium perfusion, shear stress, and the physical and chemical microenvironment that surrounds cells, and are great tools for studying biological interactions at the cellular and molecular level (Jiang et al., 2019). Recently, Meena et al. reported a microfluidic model of the BBB comprising immortalized brain endothelial cells and primary astrocytes to study immune cell transmigration (Meena et al., 2022). Several microfluidic models of the human BBB have been established in the last years aiming at physiological and biological relevance in a micro-chip with integrated physiological-like flow. While beneficial in BBB research, these models have low throughput and require pump systems for perfusion, hindering their integration with standard lab workflow and automation. To bridge this gap, we have previously reported a microfluidic model of the neurovascular unit (NVU) to study ischemic stroke in a high-throughput organ-on-a-chip platform where primary brain endothelial cells were grown in co-culture with iPSC-derived neurons and astrocytes (Wevers et al., 2021).

In the current work, we present a microfluidic model of the human BBB incorporating primary human brain endothelial cells grown under bidirectional perfusion. An inflammatory environment was mimicked in the BBBs-on-chips by exposing cultures to pro-inflammatory cytokines. To showcase the model’s potential in studying immune cell infiltration across the BBB under neuroinflammatory conditions, a T cell migration assay in presence of a chemokine trigger was used. To validate our model further, an antibody against one of the leukocyte-associated integrins was employed to assess effects on T cell adhesion and migration. The high-throughput, membrane-free, and high-content imaging capabilities of the platform make it a valuable tool for studying neuroinflammatory mechanisms and evaluation of novel drug candidates that are targeted towards reduced immune cell infiltration and BBB restoration.

## Materials and methods

### Cell culture

Primary human brain microvascular endothelial cells (HBMECs, ACBRI 376, Cell Systems) were cultured in T75 flasks (Nunc^®^ EasYFlask™, Sigma, F7552) in Promocell MV-2 medium (Bioconnect, C-22121). HBMECs were used for experiments between passage 5 and 10. THP-1 monocytes (ATCC, TIB-202™) were cultured in T25 flasks (Nunc™ EasYFlask™, Thermo Scientific, 156340) in RPMI 1640 medium (Sigma, R0883) supplemented with 10% fetal bovine serum (FBS, Sigma, F4135), 1% GlutaMAX™ (Gibco, 35050038) and 1% penicillin–streptomycin (Sigma, P4333). THP-1 cell density was maintained between 200,000 and 1,000,000 cells/mL. All cells were cultured at 37°C, 5% CO_2_ and regularly tested for mycoplasm contamination and found negative.

T cells were isolated from human peripheral blood mononuclear cells (PBMCs) that were obtained from buffy coat of a healthy donor from the Dutch blood bank (Sanquin). Methods to isolate PBMCs and T cells were previously reported (De Haan et al., 2021). Briefly, PBMCs were isolated from buffy coat by a density gradient centrifugation method using 1.078 g/mL Ficoll (GE Healthcare, 17–1440-02) and Leucosep™ tubes (VWR, 720–1840). T cells were subsequently isolated from PBMCs using the EasySep™ human T cell isolation kit (STEMCELL Technologies, 17951) according to manufacturer’s instructions. Briefly, EasySep isolation cocktail was added to PBMC suspension in AIM-V™ medium (Thermo Fisher, 12055091) and incubated at room temperature (RT) for 5 min followed by addition of RapidSpheres™. The sample was placed on a magnet (EasySep™, 18000) followed by 3 min incubation at RT. Isolated T cells were collected and maintained in a 6-well plate (Costar, 734–1599) for 48 h with DynaBeads™ Human T-Activator CD3/CD28 (Thermo Fisher, 11161D) at 1:10 bead-cell ratio for T cell stimulation.

### OrganoPlate 3-lane culture

The OrganoPlate 3-lane platform (4004-400B, MIMETAS) was used for all experiments. Lane dimensions are 400 μm × 220 μm (w × h) and phaseguides dimensions are 100 μm × 55 μm (w × h). Rat-tail collagen-I extracellular matrix (ECM) at a concentration of 4 mg/mL was prepared as previously described (Trietsch et al., 2017). Two microliter of ECM gel was dispensed into the gel inlets of the chips. After 15 min of gel polymerization in the incubator at 37°C, 50 μL Matrigel-GFR coating (80–100 μg/mL in ice cold PBS) was added to all top inlet wells. The OrganoPlate was placed back in the incubator for overnight incubation. Next, primary HBMECs at 10,000 cells/μL were seeded to the top lanes by passive pumping method (Van Duinen et al., 2019a). The OrganoPlate was incubated on its side for 3 h to allow cells to settle and attach against the gel. After 3 h of cell attachment, all medium was aspirated and 50 μL of fresh medium (OrganoMedium, HBMEC-BM, MIMETAS) was added to top inlets and outlets of each chip. Throughout the culture period, the bottom lanes were either left empty or perfused with HBSS to allow optimal barrier formation of the HBMECs in the opposite lane. The OrganoPlate was transferred to the OrganoFlow perfusion rocker (MIMETAS, OFPR-L, 7° inclination, 8 min interval) to allow HBMEC vessel formation and lumen perfusion. The rocker creates a bidirectional gravity-driven perfusion with a peak shear stress of ~1.2 dyne/cm^2^ and a peak flow rate of ~13.2 μL/min (Supplementary Figure 1). Medium changes were performed three times a week. Assays were performed between day 5 and 9 of culture.

Inflammation was induced in the model by apically exposing HBMEC tubes to varying concentrations of pro-inflammatory cytokines. TNFα (ImmunoTools, 11343015) was applied alone or in combination with IL-1β (ImmunoTools, 11340013) for 24 h. In case of combined exposures (co-exposures) of TNFα and IL-1β, both cytokines were present at the concentration reported in the figures and main text (e.g., 10 ng/mL refers to an exposure of 10 ng/mL TNFα + 10 ng/mL IL-1β in the same chip).

### T cell transmigration assay

T cells were labeled with CellTracker Orange CMRA (Invitrogen, C34551) for transmigration experiments as previously reported (De Haan et al., 2021). Briefly, T cells were incubated with 2.5 μM CellTracker Orange CMRA for 30 min. Labeling was terminated by adding AIM-V medium. The T cell suspension was centrifuged and resuspended in fresh AIM-V medium at a final density of 400,000 cells/mL. For transmigration assays, 20,000 T cells were perfused through the lumen of the endothelial vessel (top lane) followed by addition of 800 ng/mL CXCL12 (Peprotech, 300-28A) to the opposite (bottom) lane. The OrganoPlate was placed on the OrganoFlow rocker (7° inclination, 8 min interval) in the incubator (37°C, 5% CO_2_) and migration was tracked at 0, 24 and 48 h after T cell addition using the ImageXpress Micro Confocal High-Content Imaging System (Molecular Devices). For blocking experiments, T cells were pre-incubated with 50 μg/mL anti-VLA4 antibody Natalizumab (MedChem Express, HY-108831) for 45–60 min before addition to the microfluidic chips. The same concentration of Natalizumab was added to the lumen of the HBMEC vessels.

### Transendothelial electrical resistance

Methods to measure TEER in the OrganoPlate were previously reported (Nicolas et al., 2021; Wevers et al., 2021). TEER was measured by employing an automated multichannel impedance spectrometer designed for use with the OrganoPlate (OrganoTEER, MI-OT-1, MIMETAS). Prior to measurements, the OrganoTEER electrode board was disinfected with 70% ethanol and dried in a sterile flow cabinet. OrganoPlates were equilibrated to RT by placing the OrganoPlate in the flow cabinet for at least 15 min. 50 μL HBSS at RT was added to the middle inlets and outlets of each OrganoPlate chip prior to measuring TEER. TEER at baseline was determined before initiating cytokine exposure, followed by TEER measurements after 24 h cytokine exposure and 48 h transmigration studies. Data were analyzed using the OrganoTEER software that automatically extracts TEER values in Ohm*cm^2^ that are normalized to the area of the microvessel-ECM interface (estimated at 0.0057 cm^2^).

### Permeability assays

Permeability assays were performed as previously described (Trietsch et al., 2017). In short, all dry lanes of the microfluidic chip were wetted with 50 μL cell culture medium to ensure proper flow profiles. Medium was thoroughly aspirated from all inlet and outlet wells after which 20 μL fresh medium was added to the middle gel inlets and outlets, and bottom perfusion inlets and outlets. Medium with sodium fluorescein (Sigma, F6377) at a final concentration of 10 μg/mL was added to the top lanes containing the endothelial vessel (40 μL to top inlet and 30 μL to top outlet). Images were acquired from each chip every 2 min for a total duration of 12 min using an ImageXpress XLS Micro HCI System (Molecular Devices). The ratio between fluorescent signal in the middle lane (ECM gel lane) and top lane (HBMEC lane) was plotted over time to determine the leakage score (Wevers et al., 2018). Apparent permeability (P_app_) was determined by the curve fitting approach (Soragni et al., 2023).

### Immunocytochemistry

The OrganoPlate cultures were fixed with 3.7% formaldehyde (Sigma, 252,549) or 100% methanol (Sigma, 494,437). Immunostaining was performed as previously reported (Wevers et al., 2018). Briefly, cultures were permeabilized using Triton X-100 for 10 min followed by a blocking step with a buffer containing FBS, bovine serum albumin (BSA) and Tween-20 for 45 min. Primary antibodies were incubated in the blocking buffer overnight at 4°C after which secondary antibodies were incubated for 1 h at RT. The following primary and secondary antibodies were used: anti-PECAM-1 (Dako, M0823, 1:20), anti-VE-Cadherin (Abcam, ab33168, 1:1000), anti-Claudin-5 (ThermoFisher, 35-2500, 1:70, on methanol fixed cultures), anti-ICAM-1 (Bio Techne, BBA3, 1:100), anti-VCAM-1 (Abcam, ab134047, 1:100), goat-anti-mouse Alexa 555+ (ThermoFisher, A21422, 1:250), goat-anti-rabbit Alexa 488+ (ThermoFisher, A11008, 1:250), and donkey-anti-mouse Alexa 647 (ThermoFisher, A31571, 1:250). Nuclei were stained with Hoechst (H3570, ThermoFisher). Cultures were imaged with ImageXpress Micro XLS and Micro XLS-C HCI Systems (Molecular Devices).

VE-cadherin immunostainings were quantified using ImageJ software (Schindelin et al., 2012). Nuclei were counted using an automated ImageJ nuclei counting tool. VE-cadherin expression was measured by generating binary images and determining the area of the image covered by VE-cadherin staining. The area was subsequently normalized to the number of nuclei to reflect the VE-cadherin expression per cell. Junctional continuity was measured by assessing the mean length of each continuous (uninterrupted) VE-cadherin path detected using ImageJ software.

### Statistical analyses

Data were analyzed using GraphPad Prism, version 9. Data were tested for normal distribution using the Shapiro–Wilk normality test. Equality of variances were assessed. If the assumptions were not violated, one-way ANOVAs were performed. If the data assumes normal distribution but with a significant difference in the equality of variances, the Brown-Forsythe and Welch test was performed with a Dunnett T3 multiple comparisons test. If the data did not confirm normal distribution, the nonparametric Kruskall-Wallis test with Dunn’s multiple comparisons test was performed. Statistical significance was indicated by one or more asterisks. *p < 0.05, **p < 0.01, ***p < 0.001, or ****p < 0.0001.

## Results

### Lumenized vessels of brain endothelial cells form tight barriers in the OrganoPlate

**Figure 1:**
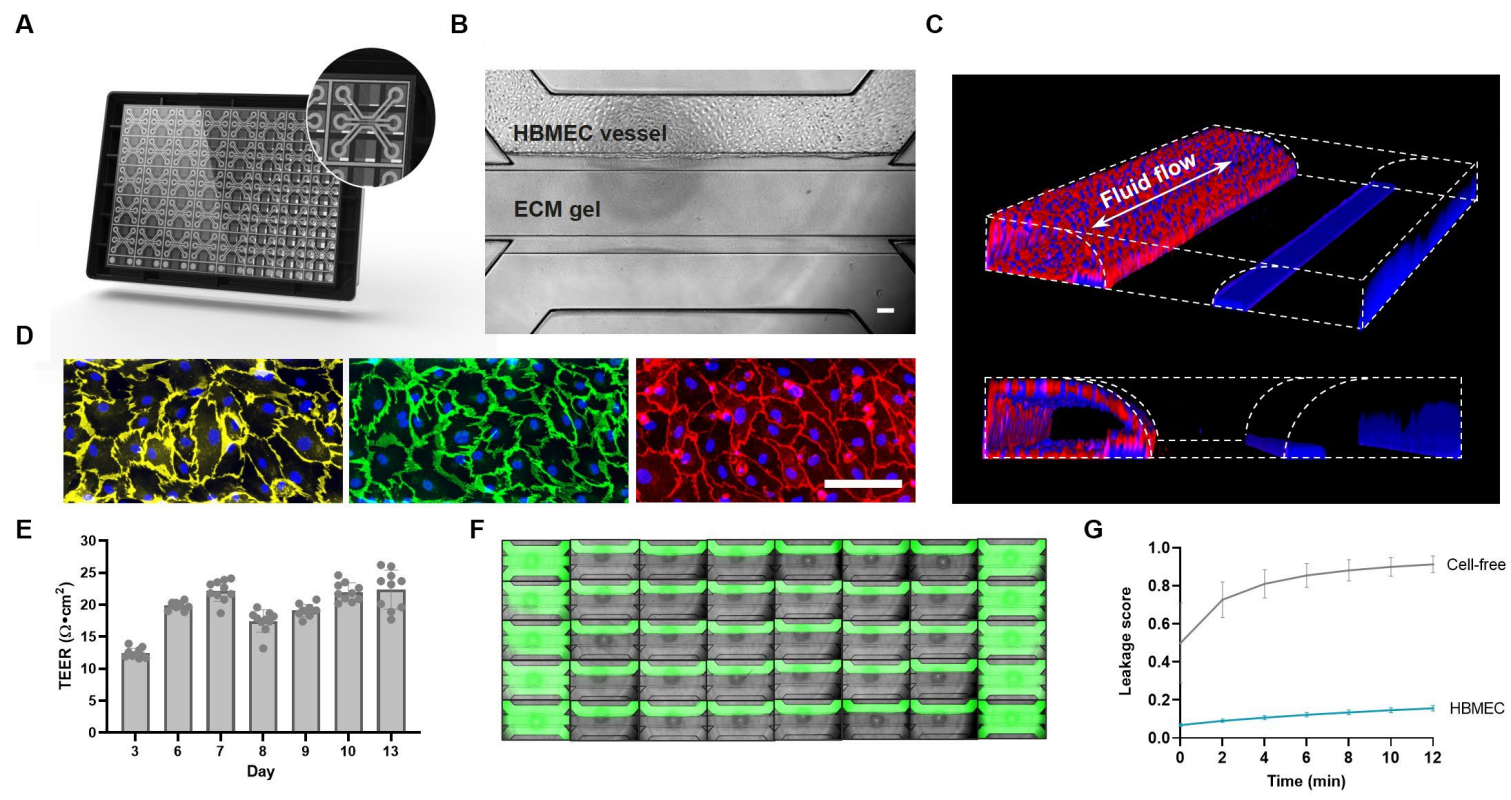
Primary brain endothelial cells form lumenized vessels against an ECM gel in the OrganoPlate and display tight barrier function. **(A)** Schematic of the OrganoPlate 3-lane platform with 40 independent microfluidic tissue culture chips. **(B)** Phase contrast image of a microfluidic chip showing HBMECs grown in the top lane against an ECM gel in the middle lane. The scale bar is 100 μm. **(C)** 3D reconstruction showing a lumenized BBB vessel stained with ActinRed and Hoechst (blue). Fluid flow is bidirectional in nature as indicated by the white arrow. **(D)** Single-plane images of HBMECs grown on the bottom of the top lane displaying expression of adherens junction proteins PECAM-1 (yellow) and VE-Cadherin (green), and tight junction protein claudin-5 (red). The scale bar is 100 μm. **(E)** BBB-on-a-chip TEER development over time, *n* = 10 chips. **(F)** Montage of a complete OrganoPlate 3-lane harboring 40 tissue chips, of which 30 chips hold a vessel of primary brain endothelial cells (middle columns) and 10 chips hold cell-free controls (first and last column). Chips were perfused with small molecule sodium fluorescein (green) to assess barrier function. Image was acquired 12 min after dye addition. **(G)** Leakage score (ratio between fluorescent signal in the ECM gel lane and HBMEC lane) of chips displayed in panel **(F)**. Graph shows mean ± standard deviation, *n* = 10–30 chips per condition.

The OrganoPlate 3-lane comprises 40 independent microfluidic chips that allow parallel culture of BBBs-on-chips for high-throughput studies. Each microfluidic chip consists of a top, middle, and bottom lane (Figure 1A). Each chip’s middle lane contains an ECM gel. This lane is patterned by PhaseGuides (Vulto et al., 2011), which prevent the gel from overflowing into adjacent lanes. Primary HBMECs are seeded in the top perfusion lane and form a lumenized microvessel under medium perfusion (Figures 1B,C). The vessels show expression of AJ proteins vascular endothelial cadherin (VE-cadherin) and platelet endothelial cell adhesion molecule (PECAM-1), and TJ protein claudin-5 at the cell–cell contacts, indicating barrier formation (Figure 1D). TEER measurements were used to assess the BBBs-on-chips’ barrier function and showed increasing TEER during the initial days of culture, followed by stable TEER up until day 13 (Figure 1E). HBMEC barrier function was also assessed using small molecule fluorescent dye sodium fluorescein (376 Da, 0.45 nm radius) (Fu et al., 1998). In contrast to cell-free control chips, BBBs-on-chips showed minimal dye leakage from their lumen with low variation, confirming tight barrier formation of the model (Figures 1F,G).

### Pro-inflammatory cytokines reduce barrier function and cause aberrant cell morphology

**Figure 2:**
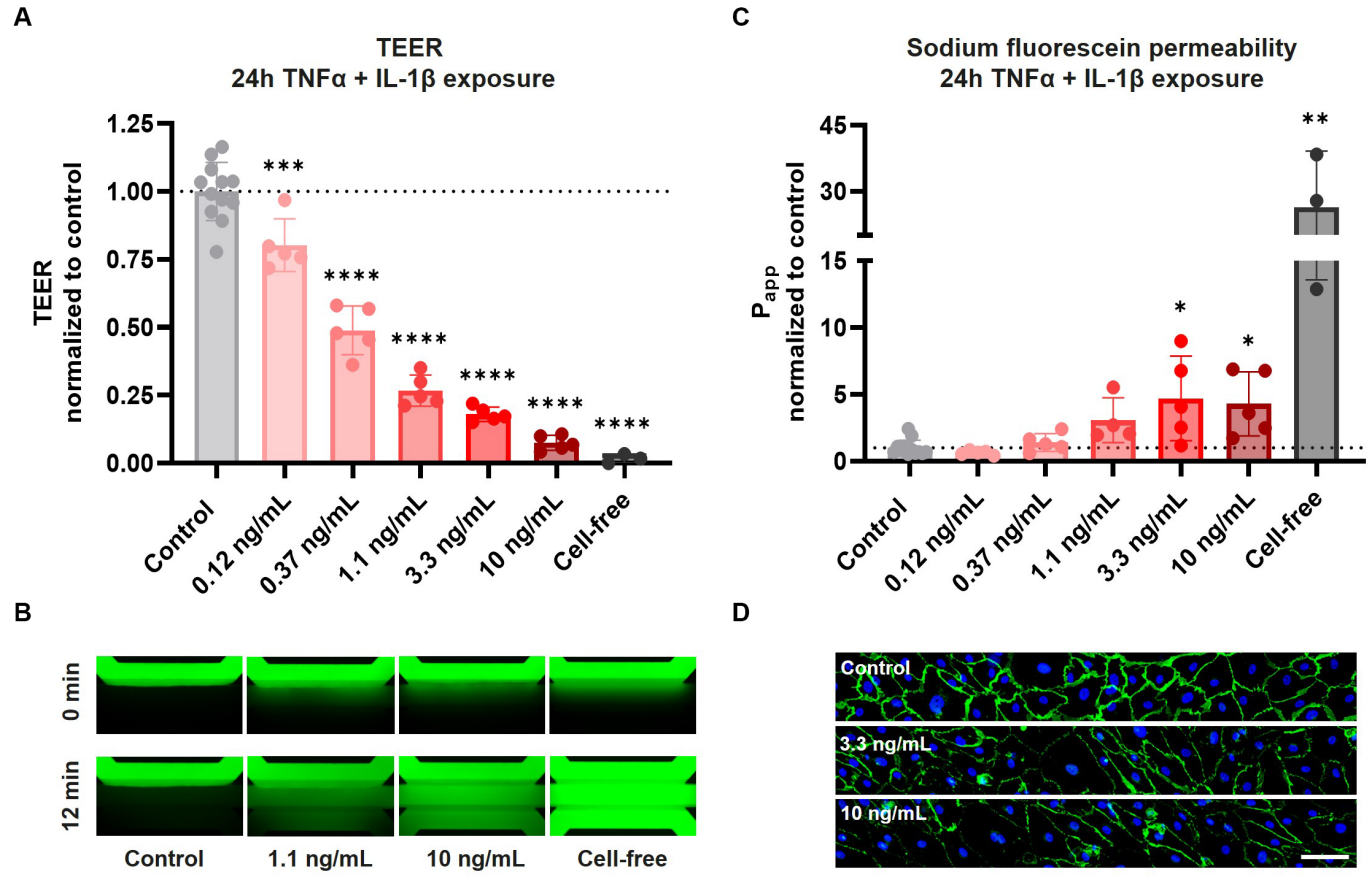
Exposure of HBMEC vessels to pro-inflammatory cytokines results in decreased barrier function and aberrant cell morphology. BBB-on-a-chip cultures were exposed to different concentrations of pro-inflammatory cytokines TNFα + IL-1β (combined in same chip) for 24 h at day 6 of culture. **(A)** TEER measurements of BBB-on-a-chip cultures normalized to medium control. Graph shows mean ± standard deviation, *n* = 3–12 chips per condition. Statistical analysis was performed using one-way ANOVA; *p < 0.05, **p < 0.01, ***p < 0.001, ****p < 0.0001. **(B)** The lumen of BBB cultures was perfused with small molecule sodium fluorescein, 376 Da (green) to assess barrier function. The panel shows representative images acquired 0 and 12 min after dye addition. **(C)** Quantification of data shown in panel **(B)**, showing apparent permeability (*P*_app_) normalized to medium control. Graph shows mean ± standard deviation, *n* = 3–12 chips per condition. Statistical analysis was performed using non-parametric Kruskal–Wallis test; *p < 0.05, **p < 0.01, ***p < 0.001, ****p < 0.0001. **(D)** BBB cultures stained for VE-cadherin (green) and Hoechst (blue) following 24 h TNFα + IL-1β co-exposure, *n* = 1 chip, scale bar: 100 μm.

After confirming AJ and TJ protein expression and barrier function in our BBBs-on-chips, cultures were exposed to a combination of pro-inflammatory cytokines TNFα + IL-1β for 24 h to mimic inflammation. Following 24 h co-exposure to TNFα + IL-1β, a concentration-dependent reduction in TEER was observed. TEER decreased up to 93 ± 3% for the highest concentration of 10 ng/mL (Figure 2A). In case of exposure to TNFα alone, a much smaller but still significant reduction in TEER was observed (up to 30 ± 6% for 10 ng/mL) (Supplementary Figure 2A).

In addition to TEER measurements, HBMEC barrier function was also assessed using sodium fluorescein. Under control conditions, barriers showed minimal leakage of sodium fluorescein, presenting with an average apparent permeability (P_app_) of 2.6 ± 1.2 × 10^−6^ cm/s (Figures 2B,C). Tight barriers were maintained following co-exposure to lower concentrations of TNFα + IL-1β (0.12–0.37 ng/mL), but significant leakage was observed following exposure to higher concentrations (3.3–10 ng/mL). Exposure to TNFα alone resulted in no significant increase in leakage of sodium fluorescein (Supplementary Figures 2B,C). These observations demonstrate that while both TEER measurements and sodium fluorescein leakage assessment show concentration-dependent barrier disruption following cytokine exposure, TEER measurements are much more sensitive in detecting alterations in barrier function.

To investigate changes in cell morphology and junctional expression following cytokine exposure, BBBs-on-chips were fixed and stained for VE-cadherin. HBMECs displayed aberrant cell morphology following co-exposure to TNFα + IL-1β, with the strongest effect observed for the highest concentrations of 3.3 and 10 ng/mL (Figure 2D, Supplementary Figure 3A). The changes in cell morphology were accompanied by reduced VE-cadherin expression and junctional continuity, but the total number of cells remained unaffected (Supplementary Figure 3B). The effects were stronger in chips co-exposed to both TNFα + IL-1β than in chips exposed to TNFα alone, in line with TEER measurements.

### Pro-inflammatory cytokines induce expression of cell adhesion molecules and increased monocyte adhesion

After assessing the BBBs-on-chips’ barrier integrity and morphology following cytokine exposure, we studied the expression of adhesion molecules on the endothelial cell surface and adhesion of THP-1 monocytes. THP-1 monocytes were labeled with Calcein-AM and perfused through the lumen of BBBs-on-chips following 24 h co-exposure to TNFα + IL-1β. Non-adherent THP-1 monocytes were washed away and cells adhered to the HBMEC vessels were quantified.

**Figure 3:**
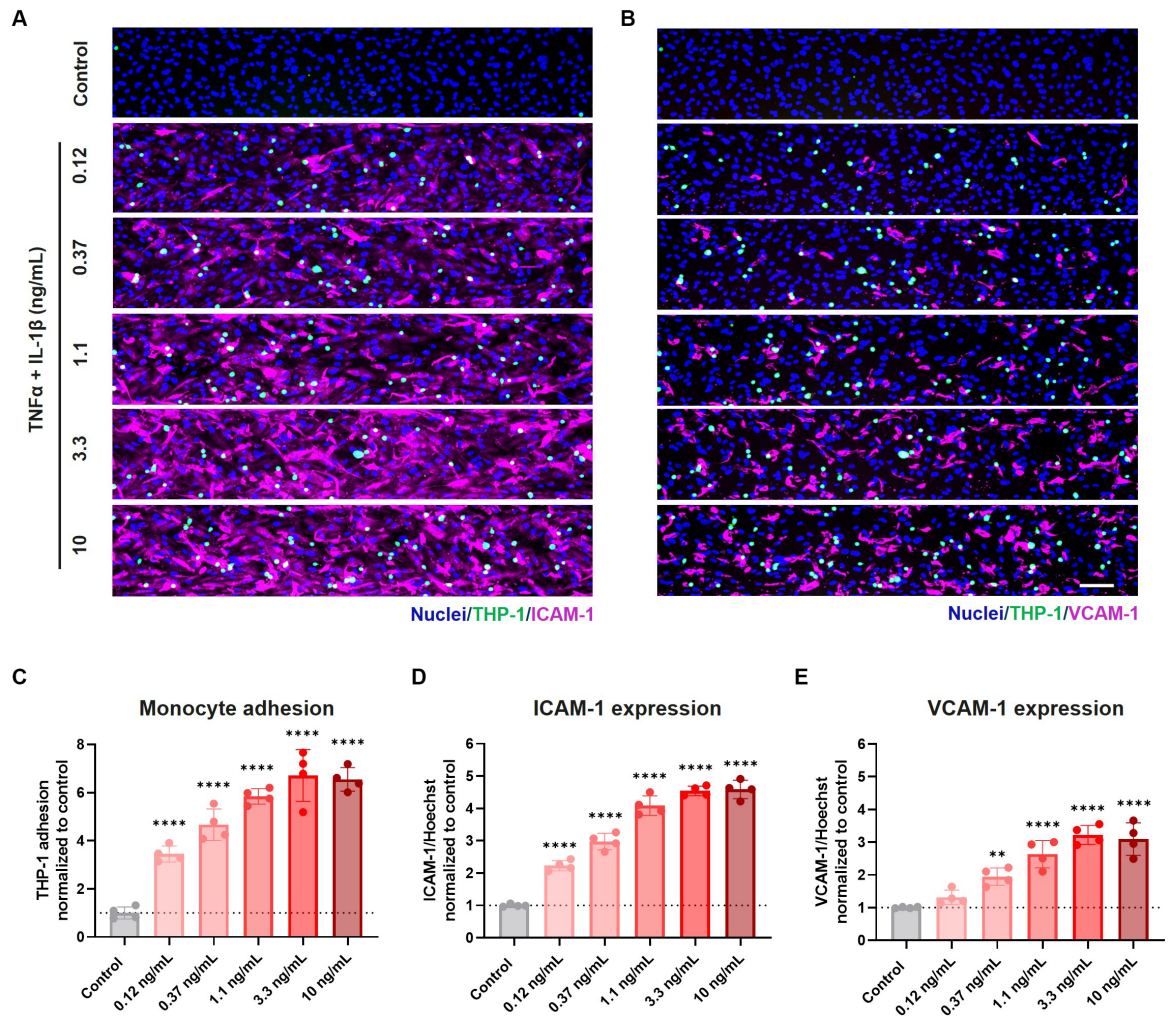
Exposure to pro-inflammatory cytokines TNFα + IL-1β results in elevated expression of cell adhesion molecules and increased monocyte adhesion to the endothelial cell surface. BBB on-a-chip cultures were exposed to different concentrations of pro-inflammatory cytokines TNFα + IL-1β (combined in same chip) for 24 h at day 6 of culture. The lumen of the cultures was perfused with fluorescently labeled THP-1 monocytes (green), after which the cultures were stained for **(A)** ICAM-1 (magenta) and Hoechst (blue), and **(B)** VCAM-1 (magenta) and Hoechst (blue). Scale bar: 100 μm. Quantification of **(C)** THP-1 monocyte adhesion, **(D)** ICAM-1 expression, and **(E)** VCAM-1 expression following varying concentrations of TNFα and IL-1β exposure. Graph shows mean ± standard deviation, *n* = 4 chips per condition. Statistical analysis was performed using one-way ANOVA; *p < 0.05, **p < 0.01, ***p < 0.001, ****p < 0.0001.

THP-1 monocytes adhered to the inflamed HBMEC vessels in a concentration-dependent manner. Monocyte adhesion was increased 3.5, 4.7, 5.8, 6.7, and 6.5-fold compared to control for increasing cytokine concentrations of 0.12, 0.37, 1.1, 3.3, and 10 ng/mL TNFα + IL-1β, respectively (Figures 3A–C). When TNFα alone was used to trigger an inflammatory phenotype, a more subtle increase was observed for low and medium range concentrations (0.37–3.3 ng/mL) (Supplementary Figures 4A–C).

Monocyte adhesion correlated strongly with the expression of cell adhesion molecules ICAM-1 and VCAM-1, whose expression showed a concentration-dependent increase following co-exposure to TNFα + IL-1β (Figures 3A,B,D,E) and to a lesser extent to TNFα alone (Supplementary Figures 4A,B,D,E).

### Primary human T cells extravasate and migrate under inflamed conditions

**Figure 4:**
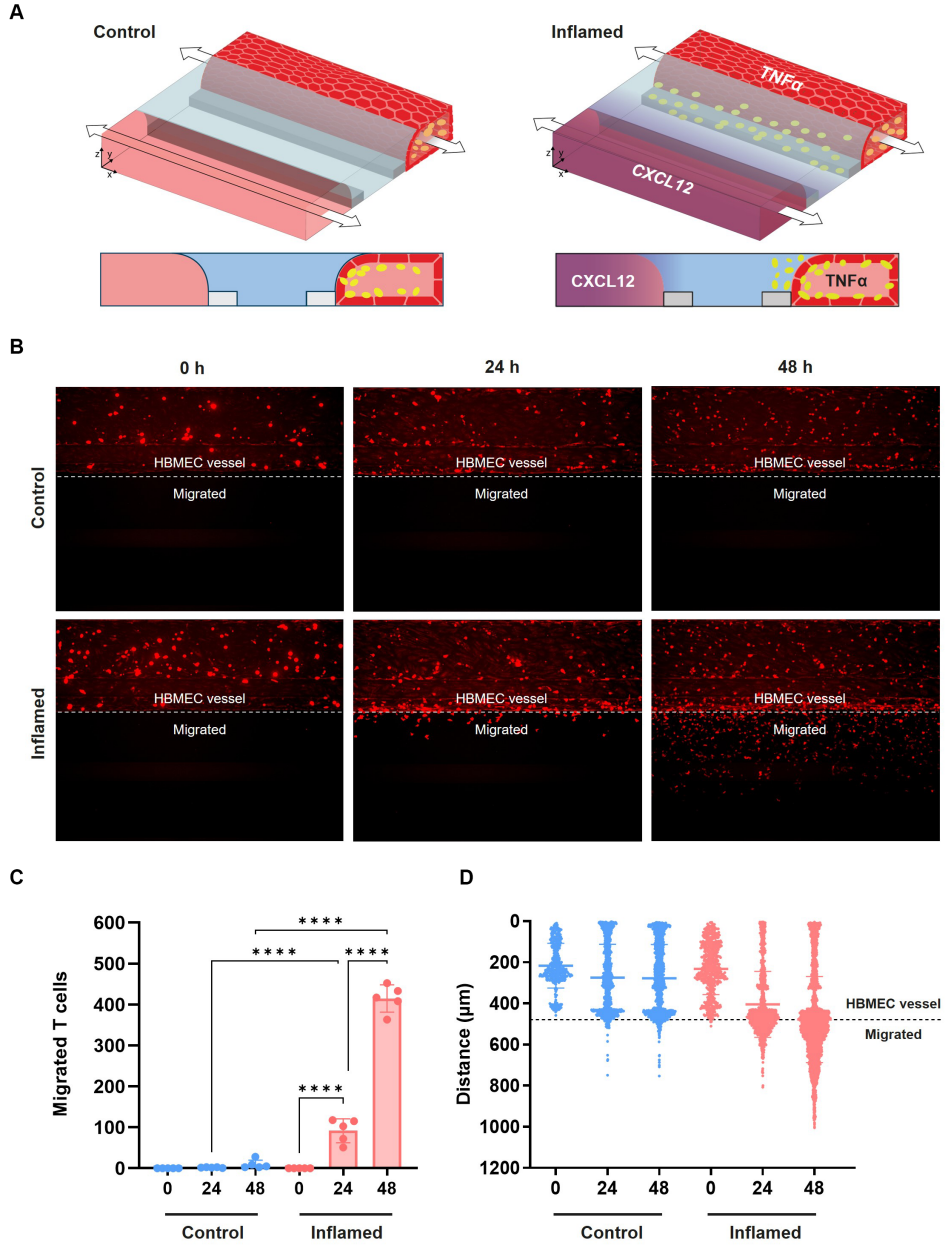
Transmigration of primary human T cells across the BBB-on-a-chip. **(A)** Schematic of T cell migration assay set-up. HBMEC vessels were exposed to control (-TNFα, -CXCL12) or inflamed (+TNFα, +CXCL12) conditions. **(B)** Representative images of T cell (red) migration under control and inflamed conditions at *t* = 0 h, *t* = 24 h, and *t* = 48 h after T cell addition. T cells were considered migrated if they were detected beyond the area of the HBMEC vessel. **(C)** Quantification of data shown in panel **(B)**, depicting T cell migration over time. Graph shows mean ± standard deviation, *n* = 5 chips per condition. Statistical analysis was performed using one-way ANOVA; *p < 0.05, **p < 0.01, ***p < 0.001, ****p < 0.0001. **(D)** Graph showing the location of fluorescently labeled T cells in the microfluidic chip of the OrganoPlate 3-lane over time under control and inflamed conditions. Each data point represents a T cell, and the location of T cells in all 5 chips per condition are displayed together in the graph. The *y*-axis depicts the distance in μm from the top of the top lane to the bottom of the bottom lane of a microfluidic chip.

After confirming the increased adhesion of the THP-1 monocyte cell line following cytokine exposure, we next assessed immune cell extravasation and transmigration across the BBBs-on-chips. Cultures were exposed to 2.25 ng/mL TNFα for 24 h to induce an inflammatory state (Supplementary Figure 4A) and subsequently perfused with fluorescently labelled stimulated primary human T cells. Chemokine CXCL12, which is known to promote T cell migration across the BBB (Calderon et al., 2006; Krumbholz et al., 2006; Li and Ransohoff, 2008), was added to the lane opposite of the HBMEC vessel. Addition of CXCL12 on the opposite side of the ECM gel results in a concentration gradient (Van Duinen et al., 2019b; De Haan et al., 2021) (Figure 4A) and is used to induce T cell migration. Migration of the T cells was assessed at 0 h, 24 h, and 48 h by confocal imaging (Figure 4B). Under control conditions (-TNFα, -CXCL12), limited T cell migration is observed towards the bottom lane of the chips over time. However, under inflamed conditions (+TNFα, +CXCL12), T cells show increased migration across the BBB-on-a-chip (Figures 4C,D). After 24 h, T cells under inflamed conditions showed 42-fold higher migration than under control conditions. After 48 h, the total number of migrating T cells increased further, maintaining a 43-fold difference between inflamed and control conditions. A 4.5-fold increase in migration was observed under inflamed conditions after 48 h when compared to 24 h with T cells having crossed the ECM gel lane and reaching the bottom lane containing the CXCL12 trigger.

### Anti-VLA4 antibody Natalizumab reduces T cell adhesion and indicates reduced migration

To validate our model further, we assessed T cell adhesion and migration in the presence of Natalizumab, an anti-VLA4 antibody therapy used for the treatment of MS. Labelled T cells were incubated with 50 μg/mL Natalizumab for 45 min and subsequently perfused through the lumen of the primary BBB-on-a-chip model. The same concentration of 50 μg/mL Natalizumab was maintained in the HBMEC vessel throughout the transmigration assay. T cell adhesion and subsequent migration were assessed 24 h, 48 h, and 72 h after addition to the BBBs-on-chips.

**Figure 5:**
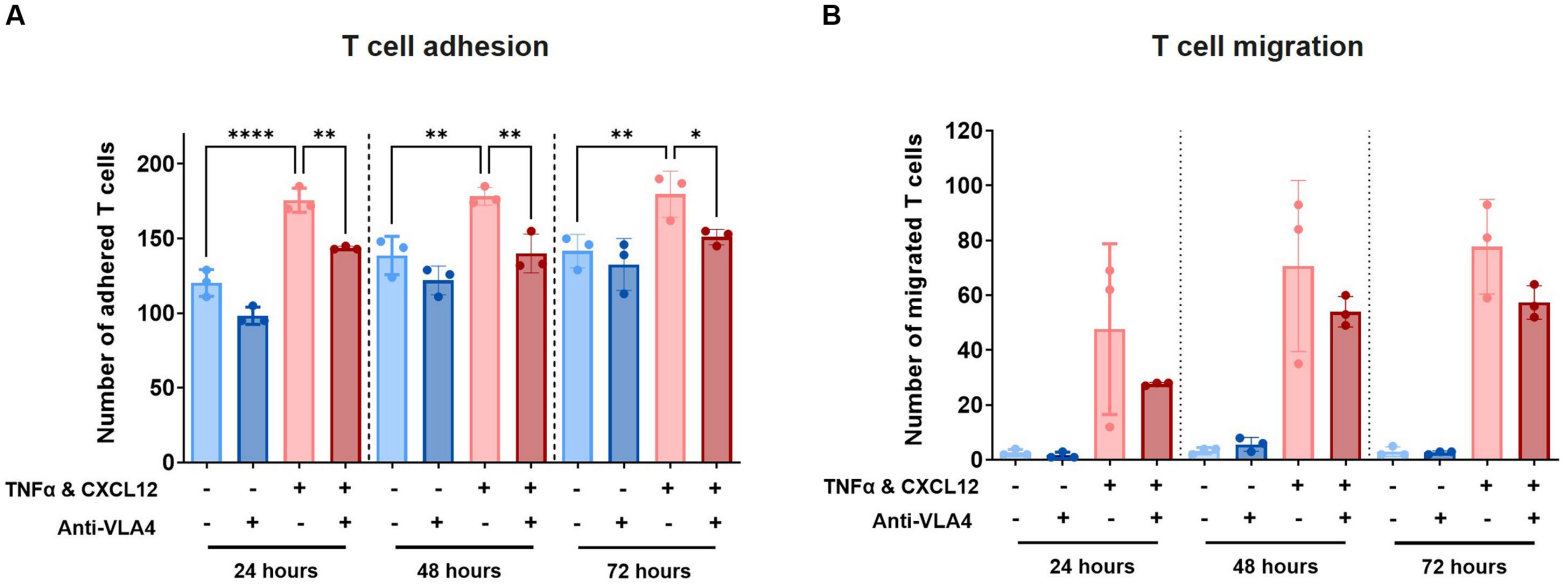
Treatment with Natalizumab results in decreased T cell adhesion and a trend toward reduced T cell migration across BBBs-on-chips. HBMEC vessels were exposed to control (-TNFα, -CXCL12) or inflamed (+TNFα, +CXCL12) conditions and perfused with primary human *T* cells in absence or presence of Natalizumab. **(A)** Number of T cells detected in the area of the chip populated by endothelial cells (adhered). Graph shows mean ± standard deviation, *n* = 3 chips per condition. Statistical analysis was performed using one-way ANOVA; *p < 0.05, **p < 0.01, ***p < 0.001, ****p < 0.0001. **(B)** Number of T cells detected outside the area of the chip populated by endothelial cells (migrated). Graph shows mean ± standard deviation, *n* = 3 chips per condition. Statistical analysis was performed using non-parametric Kruskal–Wallis test; *p < 0.05, **p < 0.01, ***p < 0.001, ****p < 0.0001.

At all tested timepoints, decreased T cell adhesion was observed in presence of Natalizumab (Figure 5A). While also observed under control conditions (-TNFα, -CXCL12), this effect was significant under inflamed conditions (+TNFα, +CXCL12). A similar trend, although not significant, was observed for T cell migration (Figure 5B). Low levels of T cell extravasation and migration were observed over time under control conditions as expected. However, under inflamed conditions (+TNFα, +CXCL12), a strong increase in T cell migration is observed compared to control (18-fold at 24 h, 21-fold at 48 h, and 26-fold at 72 h). When Natalizumab is introduced to the inflamed conditions, the observed increase in T cell migration compared to control is lower than in absence of Natalizumab (10-fold at t = 24 h, 16-fold at t = 48 h, 19-fold at t = 72 h).

## Discussion

BBB disruption, neuroinflammation, and immune cell infiltration have been reported in a range of CNS diseases. Several model systems have been established to elucidate the mechanisms of leukocyte infiltration and disease progression, but there is a need for a robust, high-throughput, and physiologically relevant platform to study the BBB in health and disease. In the current work, we developed a human BBB-on-a-chip model to study neuroinflammation and TEM. The model shows lumenized vessels of primary HBMECs against a collagen-I ECM gel and displays BBB-relevant adherens- and tight junction marker expression.

Our BBB-on-a-chip model measured an average TEER of 20–22 Ω × cm^2^ at baseline. A temporary reduction in TEER was observed on day 8 of culture, likely caused by an additional assay and medium change performed on day 7. Although it is theoretically possible to compare TEER measurements across various culture setups and systems, this is very challenging in practice as evidenced by the highly variable TEER measurements reported in studies employing identical culture platforms and culture conditions. The discrepancies in reported TEER values are even larger when different culture platforms are employed, such as Transwell versus microfluidic systems (Odijk et al., 2015; Srinivasan et al., 2015; Yeste et al., 2016). Many physical and technical parameters that influence TEER measurements differ between culture systems and setups and are challenging to control for. Among these factors are electrode configuration and placement, current distribution, temperature, membrane porosity, surface area and geometry, and computational methods (Vigh et al., 2021). For this reason, TEER values are better compared within than between studies and should be accompanied by additional methods to assess barrier function. We used small molecule sodium fluorescein (376 Da) to support our TEER measurements and observed near-complete retention of the dye within the lumen of the BBBs-on-chips. With an average P_app_ of 2.6 ± 1.2 × 10^−6^ cm/s, the leakage is within the P_app_ range reported for rat brain microvessels *in vivo* (Hawkins et al., 2007; Yuan et al., 2009). The combination of the two assays confirmed tight baseline barrier function in our model. Both assays detected concentration-dependent BBB disruption following exposure to pro-inflammatory cytokines, with TEER measurements being much more sensitive, as previously reported (Gijzen et al., 2020; Nicolas et al., 2021).

In addition to the effect on barrier function, exposure to pro-inflammatory cytokines results in brain endothelial cell activation and enhanced expression of CAMs (Tsukada et al., 1993; Afonso et al., 2007; Man et al., 2012; Spampinato et al., 2021). In our BBB model, an aberrant endothelial cell morphology with a significant loss of BBB function was observed after 24 h cytokine treatment, especially at higher concentrations. This change in cell morphology is likely caused by the occurrence of endothelial to mesenchymal transition (endoMT). Studies have shown that endoMT may be induced by pro-inflammatory cytokines such as transforming growth factor β (TGF-β), TNFα, and IL-1β, which cause endothelial cells lose their specialized BBB function and dedifferentiate to mesenchymal cells (Troletti et al., 2016; Cho et al., 2018; Derada Troletti et al., 2019; Sun et al., 2022).

Following cytokine exposure, HBMECs displayed significantly increased expression of key cell adhesion molecules ICAM-1 and VCAM-1 in a concentration-dependent manner. Enhanced CAM expression is known to play a crucial role in immune cell recruitment, adhesion, and migration across the BBB (Marchetti and Engelhardt, 2020). Studies have reported differences in the kinetics of ICAM-1 and VCAM-1 upregulation following cytokine stimulation of HBMECs (Wong and Dorovini-Zis, 1992; Wong and Dorovini-Zis, 1995; Lee et al., 2017). The expression levels may be impacted by the specific cytokine, the concentration, but also the type of endothelial cell used. Additionally, post-stimulation levels of ICAM-1 are reported to be much higher compared to VCAM-1, indicating that fewer cells express VCAM-1 compared to ICAM-1 *in vitro* (Wong and Dorovini-Zis, 1992; Wong and Dorovini-Zis, 1995). This is in line with our observation of a slightly higher and more homogenous expression pattern of ICAM-1 as opposed to a lower and sparser VCAM-1 expression. As expected, we observed a strong correlation between ICAM-1 and VCAM-1 expression and THP-1 monocyte adhesion.

Previous studies performed in our laboratories modeled vascular inflammation using TNFα (Poussin et al., 2020; Ehlers et al., 2023). In the present study, we observed that exposing the BBBs-on-chips to a combination of TNFα + IL-1β led to increased barrier disruption, endothelial CAM expression, and monocyte adhesion compared to exposure to TNFα alone. Because our experiments did not investigate the effect of IL-1β alone, we cannot extract the individual contribution of each cytokine to our findings in the TNFα + IL-1β co-exposed condition. While it is theoretically possible that the observed effects are a mere summation of each cytokine’s individual impact, it is more likely that these two cytokines synergistically complement each other, leading to an enhanced response greater than the sum of their individual effects (Stahl et al., 2003). It may also be noted that TNFα and IL-1β can differentially induce changes in barrier permeability (Versele et al., 2022). Furthermore, an increased expression of ICAM-1 and VCAM-1 on brain ECs was reported with TNFα exposure when compared to IL-1β (O’carroll et al., 2015).

For T cell migration studies, BBBs-on-chips were pre-exposed to TNFα alone according to previously optimized protocols and T cell migration was tracked under the influence of a CXCL12 chemokine trigger (De Haan et al., 2021). CXCL12 is a highly potent chemokine for attracting immune cells, especially lymphocytes and monocytes in the context of CNS diseases (Calderon et al., 2006; Krumbholz et al., 2006; Li and Ransohoff, 2008; Liu and Dorovini-Zis, 2009). Addition of CXCL12 to the basolateral side of our model enabled chemotaxis, allowing directional transmigration of T cells along a concentration gradient. A small increase in T cell migration was already observed after 6 h of perfusing T cells (data not shown) but a significant increase in migration occurred after 24 h, with a large number of T cells migrating to the basolateral compartment by 48 h. In contrast, T cell migration under control conditions was minimal. For T cell quantification, we tested various threshold settings to balance false positives and ensure the detection of true positives. While the selected approach slightly overestimates T cell migration, especially noticeable under control conditions with few migrating T cells, it still maintains an acceptable signal-to-noise ratio, accurately reflecting T cell migration differences across experimental conditions. To further validate our model, we investigated the effects of anti-VLA4 monoclonal antibody Natalizumab on T cell adhesion and migration. Natalizumab treatment significantly reduced T cell adhesion to inflamed BBBs-on-chips and showed a trend towards reduced migration. Studies on MS patients have reported that T cells switch to alternate modes of adhesion and migration under continuous Natalizumab therapy as a compensatory mechanism (Wipfler et al., 2011; Schneider-Hohendorf et al., 2014). Additionally, Natalizumab was shown to only partially and transiently reduce α4-integrin-mediated firm T cell adhesion to TNFα-activated micro-vessels (Coisne et al., 2009). Furthermore, while T cell adhesion is mediated by VLA-4 and LFA-1 interactions with their respective endothelial counterparts VCAM-1 and ICAM-1, migration is strongly dependent on ICAM-1 and ICAM-2 (Coisne et al., 2009; Steiner et al., 2010; Rothhammer et al., 2011). Taken together, alternate modes of adhesion and migration are assumed by T cells with Natalizumab treatment explaining why a total abrogation of adhesion and migration was not observed in our model. Blocking other ligands and adhesion molecules alone or in combination with VLA-4 in our model may provide deeper insights into T cell migration and blocking mechanisms.

The peak shear stress employed in the BBBs-on-chips in this study is approximately 1.2 dyne/cm^2^. This falls in the range reported for post-capillary venules (1–6 dyne/cm^2^ for 20–50 μm vessel diameters) and is thus relatively low compared to vessels of comparable diameter (300–400 μm) *in vivo* (Dewey et al., 1981; Cucullo et al., 2002; Chiu and Chien, 2011). Furthermore, unlike *in vivo*, the flow in our BBBs-on-chips is of a bidirectional nature. More physiological flow can be obtained in microfluidic systems by employing pump-based perfusion rather than gravity-driven perfusion (Destefano et al., 2017; Park et al., 2019; Ahn et al., 2020). However, this comes at the cost of diminished throughput and ease of use, which are well known challenges in adopting microfluidic models in routine experimentation (Dewey et al., 1981; Cucullo et al., 2002; Chiu and Chien, 2011). Although our model is suboptimal for research questions requiring full control of flow patterns, bidirectional flow still holds advantages over static cultures. It has been reported that shear stress associated with flow enables endothelial cells to incorporate and maintain BBB characteristics *in vitro* (Man et al., 2012; Takeshita and Ransohoff, 2012; Wevers et al., 2018, 2021). Additionally, dynamic *in vitro* models have shown that flow is crucial in leukocyte transmigration across cellular barriers (Cinamon et al., 2001a,b; Schreiber et al., 2007; De Haan et al., 2021).

Type I collagen ECM was used as a scaffold matrix to model BBBs-on-chips. Although the collagen-I concentration in the brain is very low (Ferro et al., 2020), a wide variety of *in vitro* BBB models have favored the use of this hydrogel (Herland et al., 2016; Partyka et al., 2017; Wevers et al., 2018; Linville et al., 2019; Shin et al., 2019). This is mainly due to its mechanical strength, improved cell-matrix adhesion and remodeling, ease of availability, and relatively lower costs (Kim et al., 2015; Van Der Helm et al., 2016; Ferro et al., 2020). However, different lots of collagen hydrogel may be highly variable and the ECM properties depend on the fabrication parameters, including collagen source or gelation pH (Antoine et al., 2014). Thus, ECM characterization is a recommended prerequisite for *in vitro* BBB modeling. The stiffness of the ECM may also be considered for immune cell transmigration studies. In the current work, 4 mg/mL rat-tail collagen-I resulted in stable barrier formation without cell invasion while enabling successful T cell transmigration.

Our BBB-on-a-chip model incorporates primary brain endothelial cells. Although primary cells offer relatively close resemblance to their *in vivo* counterparts, these cells may dedifferentiate and lose tissue-specific characteristics upon isolation from their *in vivo* environment. Moreover, donor-to-donor and batch-to-batch variability may be kept in mind while using primary cells (Gumbleton and Audus, 2001; Terasaki et al., 2003; Helms et al., 2016). In response to this gap, protocols for the generation of iPSC-derived brain endothelial cells (iBECs) were reported. While the resulting cultures show very tight barrier function and expression of BBB-relevant markers, studies have reported that iBECs also display a strong epithelial phenotype (Vatine et al., 2019; Lu et al., 2021). This is in line with our experience with iBECs in the OrganoPlate (data not shown). Hence, we employed primary brain endothelial cells in our BBB model. We have worked with three different donors without observing significant variability but cells from only one donor were used in the current study for consistency.

The model presented here consists of HBMECs only. This monoculture model allows for short culture timelines and robust barrier formation, enabling high-throughput studies of the endothelial response to inflammatory conditions and T cell migration across the endothelial barrier. Future studies may investigate neuroinflammation in more complex co-culture models of the NVU by incorporating supporting cells such as astrocytes, pericytes, neurons, and microglia in our chips (Koo et al., 2018; Wevers et al., 2018, 2021; Verma et al., 2022). Furthermore, instead of employing exogenous cytokines to induce inflammation as we have done in the current study, conditioned medium collected from leukocytes containing soluble factors or M1 and M2 microglia conditioned media may be used.

In conclusion, we report a primary human BBB on-a-chip model in a high-throughput microfluidic platform that is compatible with standard laboratory equipment and automation. The model presented here will aid in studying neuroinflammation at the BBB and assessment of potential restorative therapies.

## Data availability statement

The original contributions presented in the study are included in the article/Supplementary material, further inquiries can be directed to the corresponding author.

## Ethics statement

Ethical approval was not required for the studies on humans in accordance with the local legislation and institutional requirements because only commercially available established cell lines were used.

## Author contributions

ALN, NRW, and HEV conceptualized and designed the study and wrote the manuscript with input from all authors. ALN, LG, RO, TMF, RA, and OM performed experiments and data analysis. NRW and HEV supervised the study. All authors approved the submitted version.

## Funding

This work was supported by the Marie Sklodowska-Curie International Training Network ENTRAIN (Grant Agreement #813294) and the Innovative Medicines Initiative 2 program IM2PACT (Grant Agreement #807015).

## Conflict of interest

The authors declare that the research was conducted in the absence of any commercial or financial relationships that could be construed as a potential conflict of interest.

## Publisher’s note

All claims expressed in this article are solely those of the authors and do not necessarily represent those of their affiliated organizations, or those of the publisher, the editors and the reviewers. Any product that may be evaluated in this article, or claim that may be made by its manufacturer, is not guaranteed or endorsed by the publisher.
