# Supplementary figures and images for: Human BBB-on-a-chip reveals barrier disruption, endothelial inflammation, and T cell migration under neuroinflammatory conditions
Source: Front Mol Neurosci. 2023 Sep 25;16:1250123. doi: 10.3389/fnmol.2023.1250123 (PMC10561300; doi:10.3389/fnmol.2023.1250123)

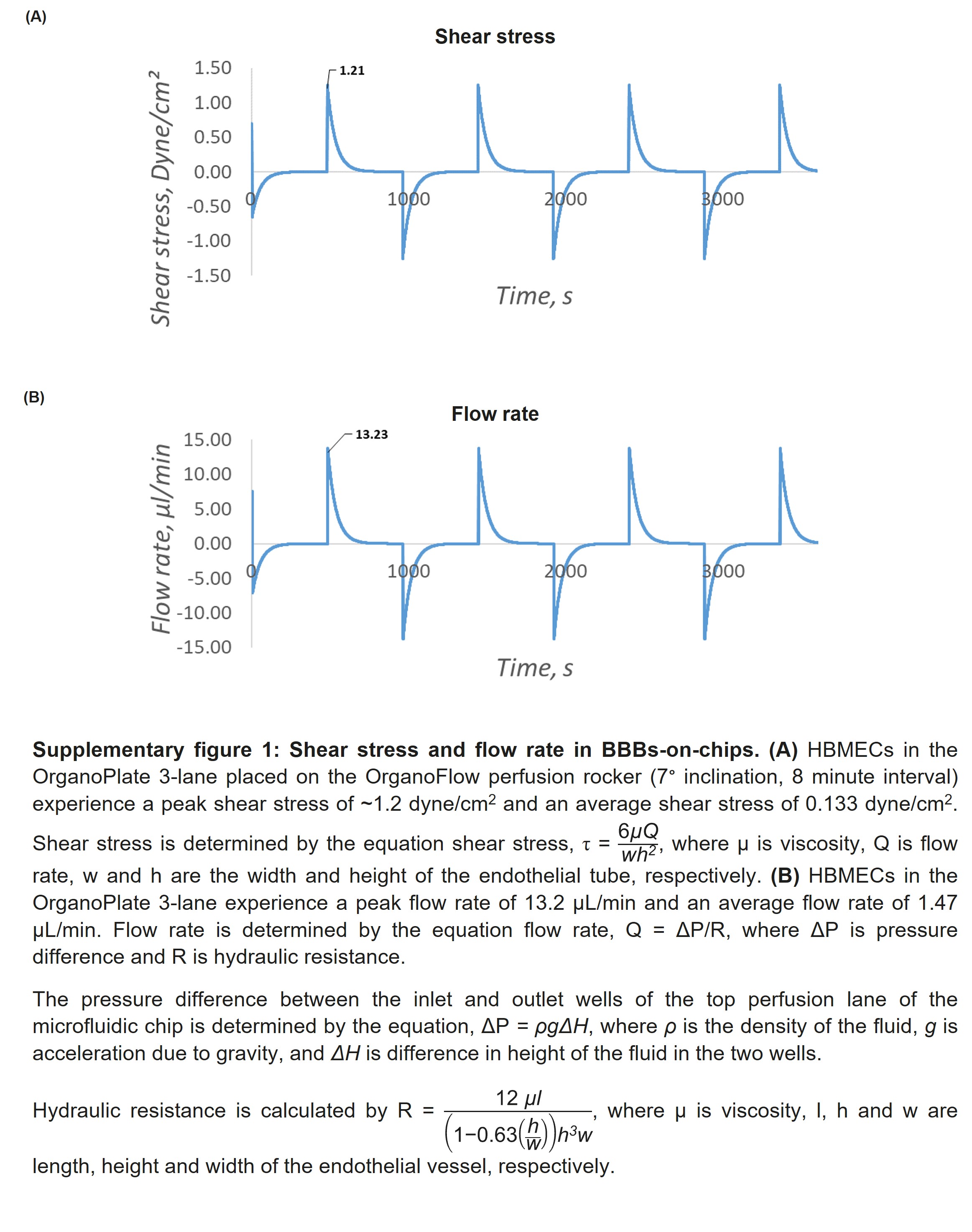

Supplement: Supplementary file 1 [file Image_1.JPEG]

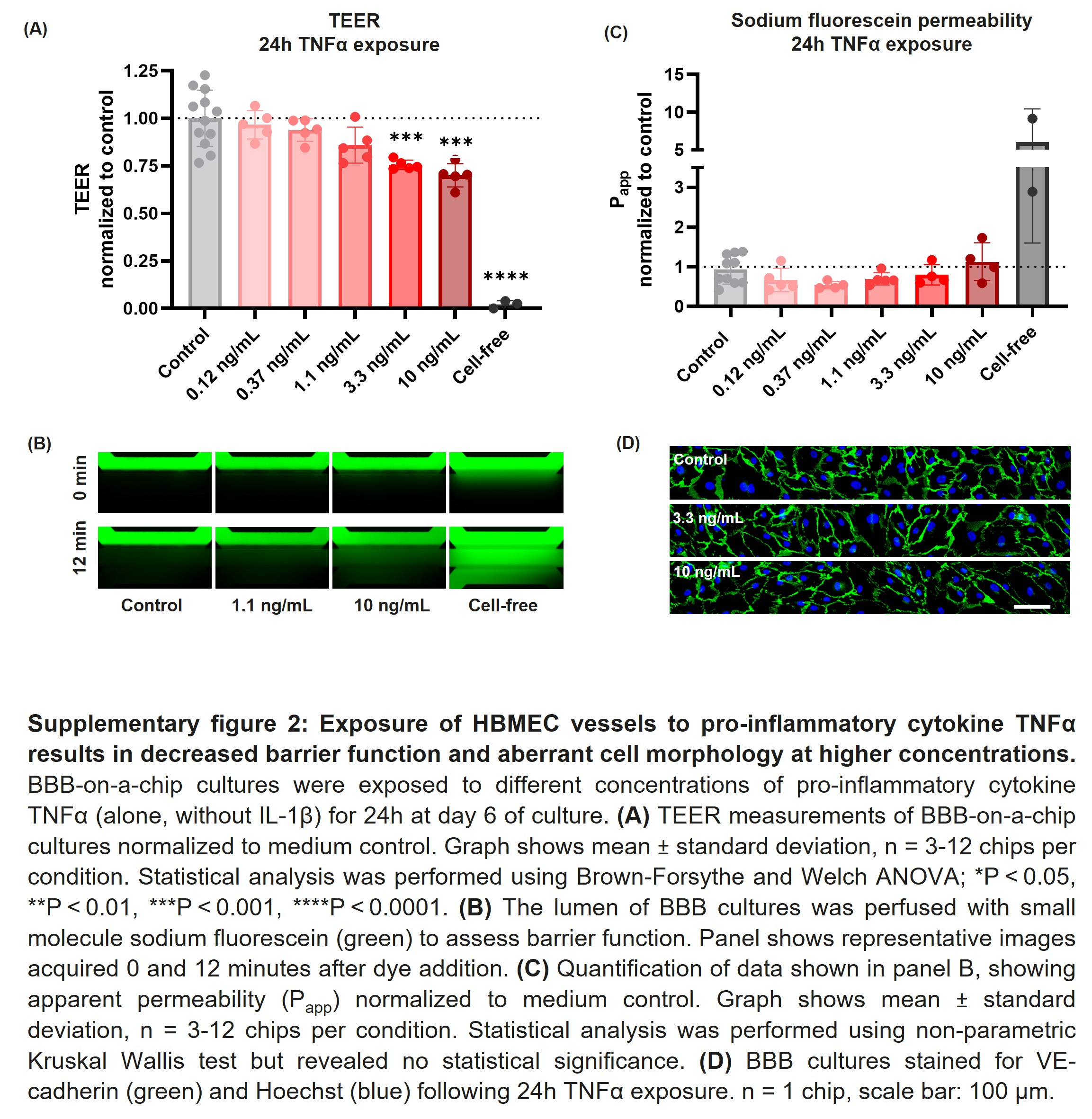

Supplement: Supplementary file 2 [file Image_2.JPEG]

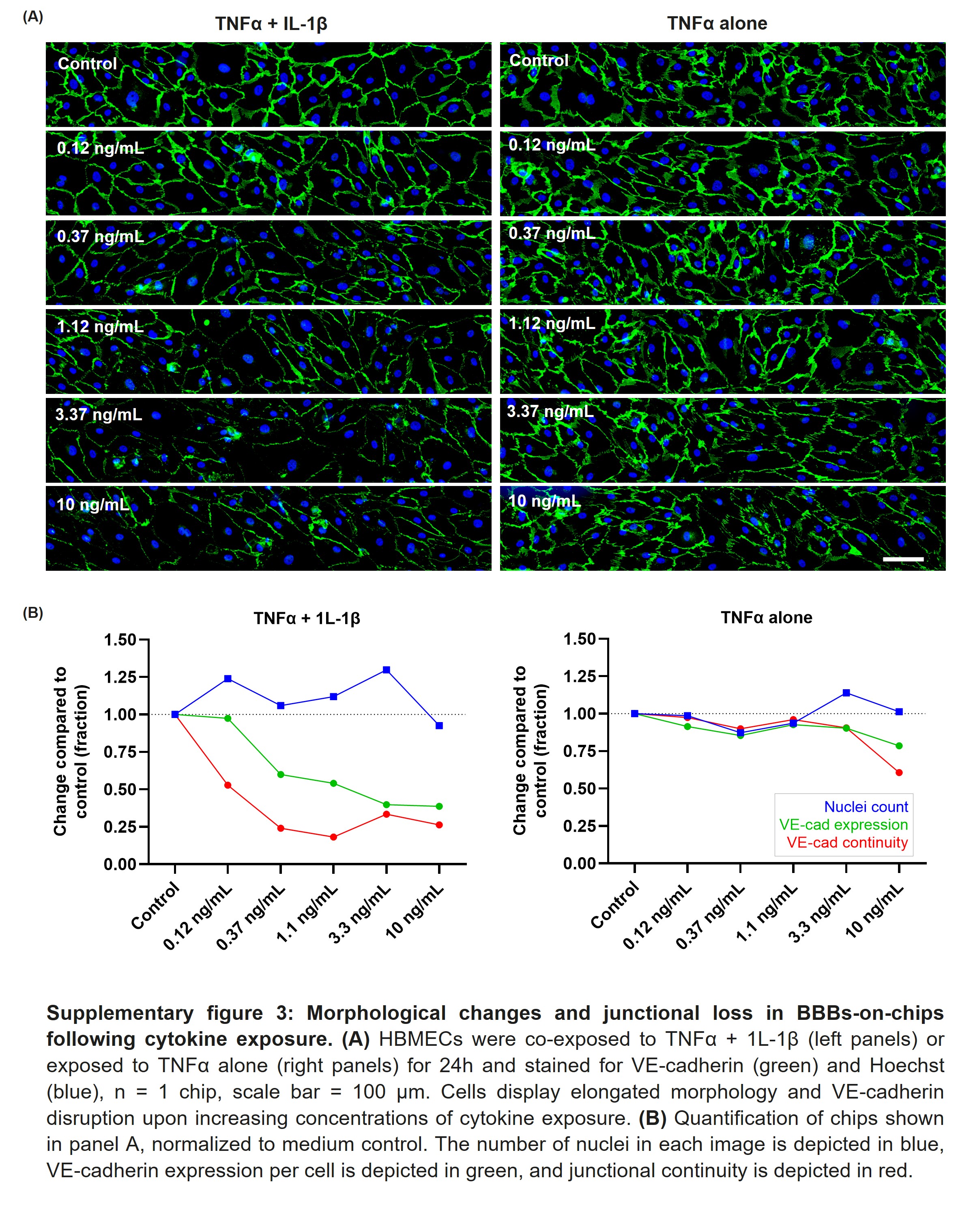

Supplement: Supplementary file 3 [file Image_3.JPEG]

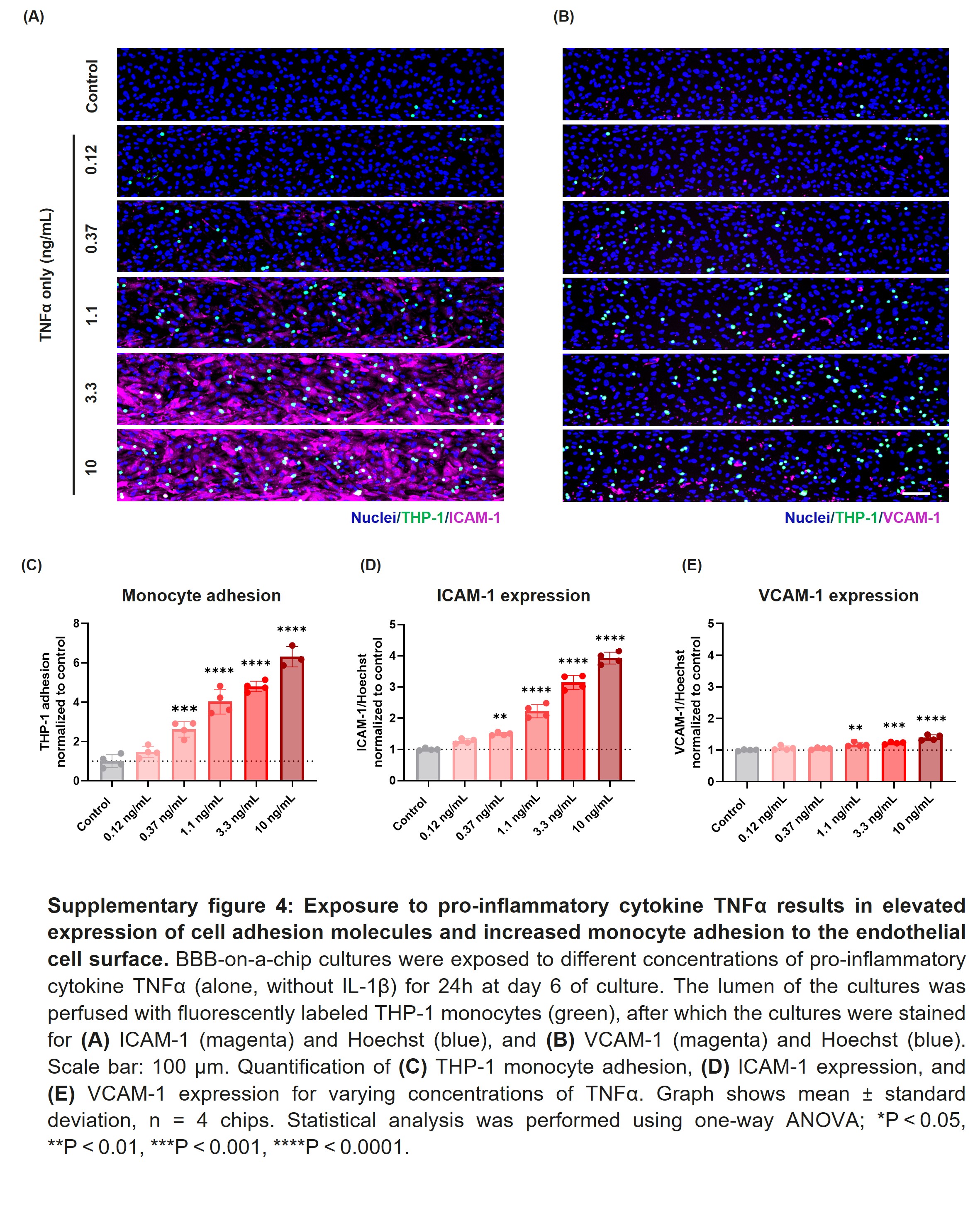

Supplement: Supplementary file 4 [file Image_4.JPEG]
